# Attention Deficit/Hyperactivity Disorder and Risk of Dementia: A Systematic Review and Meta-Analysis

**DOI:** 10.3390/brainsci16060646

**Published:** 2026-06-18

**Authors:** Ludovico Baiamonte, Giovanna Bellante, Patrizio Allegra, Domenico Tarantino, Claudia Migliazzo, Manuela Lodico, Laura Maniscalco, Tommaso Piccoli, Nicola Vanacore, Domenica Matranga, Giuseppe Salemi

**Affiliations:** 1 Department of Diagnostic, Interventional and Stroke Radiology, UOC Neurology, AOUP ‘P. Giaccone’, 90100 Palermo, Italy; 2 Department of Biomedicine, Neuroscience and Advanced Diagnostics, University of Palermo, 90100 Palermo, Italy; 3 Department of Health Promotion, Mother and Child Care, Internal Medicine and Medical Specialties, University of Palermo, 90100 Palermo, Italy; 4 National Center for Disease Prevention and Health Promotion, Italian National Institute of Health, 00100 Rome, Italy

**Keywords:** attention deficit/hyperactivity disorder, dementia, systematic review, meta-analysis, risk factors, epidemiology

## Abstract

Introduction: The significant impact of attention deficit/hyperactivity disorder (ADHD) on health in adult life has been widely recognized. Among the comorbidities of this disorder in later life, dementia is one of the most relevant ones. We performed a systematic review and meta-analysis to explore the impact of previous ADHD diagnosis on dementia risk. Materials and methods: We systematically searched Pubmed, Embase and Scopus for the relevant literature. Cohort and case–control studies were included in our review. Retrieved records were selected by title and abstract and then by full text reading. For quality appraisal, the Newcastle–Ottawa scale was used. A meta-analysis of hazard ratios (HRs) was performed for each type of dementia. Results: Four cohort studies and one case–control study were included, for a total of 3,703,877 and 400 participants, respectively. For all-cause dementia, the pooled HR was 2.52 (95%CI 1.51–4.22, *p* < 0.001), pointing out a significantly higher hazard in subjects with ADHD. For Alzheimer’s disease, vascular dementia and Lewy body dementia, no meta-analysis was performed due to the low number of available studies. Discussion and conclusions: Our results support a significant association between ADHD and risk of dementia. The results regarding specific types of dementia are more challenging to interpret and could have been influenced by sample size issues. These findings show that ADHD deserves attention in future research on cognitive disorders of the elderly; in particular, more studies are needed to reveal if a true causal relationship links ADHD and dementia.

## 1. Introduction

Dementia is a rapidly growing public health problem, affecting about 50 million people all over the world. There are nearly 10 million new cases every year and this figure is set to triple by 2050 [1]. The most common type of dementia is Alzheimer’s disease (AD), accounting for about 60% to 70% of dementia cases, followed by vascular dementia (VaD) with a prevalence of about 20% [2]; other types of dementia include dementia with Lewy bodies (DLB), Parkinson’s disease dementia (PDD), and frontotemporal dementia (FTD) [3]. While there is no curative treatment for dementia, the proactive management of modifiable risk factors can delay or slow the onset or progression of the disease [1]. Attention deficit/hyperactivity disorder (ADHD) may be a risk factor for the later development of a neurodegenerative disease or dementia, as highlighted by the study by Becker et al. published in 2023 [4].

ADHD is a psychiatric condition that has long been recognized as affecting children’s ability to function. Individuals suffering from this disorder show patterns of developmentally inappropriate levels of inattentiveness, hyperactivity, or impulsivity. The Diagnostic and Statistical Manual of Mental Disorders, Fifth Edition, Text Revision (DSM-5-TR) provides a set of criteria for the diagnosis of this disorder: symptoms begin at a young age and usually include lack of attention, lack of concentration, disorganization, difficulty completing tasks, being forgetful, and losing things. In order to be labeled as ADHD, these symptoms should be present before the age of 12 and in more than one setting (i.e., at home, school, or after-school activities), have lasted for at least six months, and interfere with daily life activities. ADHD can have broad consequences, including social interactions, increased risky behaviors, loss of jobs, and difficult scholastic achievements [5]. Even if it has been initially described in children and it is usually diagnosed in pediatric age, the importance of ADHD extends across the entire lifespan, since it is estimated that it persists into adulthood in as many as 90% of those diagnosed as children and that its prevalence in adults is about 2.8% [6]. This is relevant because adult ADHD is linked to higher risk of social and educational inequalities, substance abuse, sleep disorders, cardiovascular diseases and mental health problems [6].

Regarding the burden of ADHD in later age, dementia deserves special attention, due to its pervasive consequences on patients and their caregivers. The higher risk of dementia in ADHD subjects that has been observed in previous studies could be the result of multiple mechanisms, such as the existence of common predisposing factors at genetic and neurobiological levels, lower cognitive reserve and consequent greater susceptibility to the effects of neurodegeneration and increased exposure to risk factors for dementia. Previous reviews have addressed the potential link between ADHD and dementia; however, in existing papers this theme was framed in a broader perspective, since these reviews included other neurodegenerative disorders [4] or studies with other designs [7], giving rise to heterogeneity that prevented authors from any quantitative pooling of collected evidence. Moreover, they summarized available evidence up to 2023, whereas the relevant literature has kept on expanding after this date.

Due to the constant growth of available evidence regarding the impact of ADHD on the risk of developing dementia, we performed a systematic review and meta-analysis on this topic.

## 2. Materials and Methods

We performed a systematic review and meta-analysis and reported our findings according to the Preferred Reporting Items for Systematic Reviews and Meta-Analyses (PRISMA) guidelines [8,9] (see Table S1 in Supplementary Material for the PRISMA checklist). Our review was registered on the International Prospective Register of Systematic Reviews (PROSPERO; CRD420251059343).

### 2.1. Data Sources and Search Strategy

We systematically searched MEDLINE (via PubMed), Embase (via Ovid) and Scopus databases from inception to 16 May 2025. Polyglot tool [10], part of the Systematic review accelerator (SRA) online platform [11], was used to assist us in string conversion between different sources. The search strings are included in Tables S2–S4 in the Supplementary Materials. The reference list of studies selected for inclusion and published systematic reviews on the same topic were also screened for studies that met our inclusion criteria. In the following search, the retrieved titles were collated in Zotero software (version 6.0.36), then duplicated items were removed using the Deduplicator tool [12] of the SRA. Screening by title and abstract was performed by two reviewers (GB and PA) using Rayyan platform [13]; disagreements were solved by discussion between the two authors and, if needed, through the involvement of a third one. The same method was used for full-text screening.

### 2.2. Eligibility Criteria

We included studies: (1) adopting a case–control or cohort design; (2) quantitatively exploring the association between ADHD and dementia through the use of adequate effect measures; (3) defining ADHD according to standardized criteria, such as those of the Diagnostic and Statistical Manual of Mental Disorders (DSM) or the International Classification of Diseases (ICD) coding system; (4) defining dementia according to clinical and/or biomarker-based diagnosis (a broad type of dementia/mild cognitive impairment was considered, including, but not limited to, Alzheimer’s dementia, frontotemporal dementia, alpha-synucleinopathies with dementia, motor tauopathies with dementia, vascular dementia); (5) including at least five exposed participants in each group; (6) published in peer-reviewed journals; and (7) written in the English language.

We excluded: (1) book chapters, reviews, letters, case reports or conference abstracts; (2) studies not providing enough data to calculate the risk estimates; and (3) studies on animal models.

### 2.3. Data Extraction

Data extraction was performed using an electronic sheet. To extract data from plots, Plotdigitizer tool was used [14]. Extracted data for each study regarded publication year, region, study design, sample size, mean or median age of participants overall and in each group, gender of participants overall and in each group, ADHD ascertainment method, ADHD definition, ADHD severity, age at ADHD diagnosis, dementia type, age at dementia diagnosis, effect size and its variance, and effect size adjustment.

### 2.4. Outcomes

The main outcome was the onset of dementia, including AD, FTD, alpha-synucleinopathies with dementia, and motor tauopathies with dementia and VaD. Secondary outcomes included evaluation of the risk of bias of included studies and exploration of possible sources of heterogeneity.

### 2.5. Quality Appraisal

Assessment of risk of bias of the included studies was performed using the Newcastle–Ottawa Scale (NOS) [15]. As required by this tool, individual studies were assessed based on three quality parameters: selection, comparability, and outcome.

### 2.6. Data Synthesis and Analysis

For each type of dementia, effect measures of association between ADHD and risk of dementia were odds ratio (OR) for case–control studies and hazard ratio (HR) for retrospective cohort studies. Homogeneous statistics were pooled using a random-effects model. Two-sided *p* values of 0.05 or less were considered statistically significant unless otherwise stated and the individual and pooled effect sizes were shown using a forest plot. The statistical heterogeneity among the studies was assessed by the Cochran Q statistic (*p* values < 0.10 will be considered indicative of statistically significant heterogeneity) and I^2^ statistic (I^2^ values less than 25% represent mild heterogeneity, values between 25% and 50% represent moderate heterogeneity, and values greater than 50% represent large heterogeneity).

To investigate possible sources of heterogeneity among the included studies, sensitivity analyses and meta-regression analyses were planned. Moreover, we used leave-one-out analysis to look for influential studies and re-calculated summary estimates after removing them. We also planned to assess publication bias using a funnel plot with Egger’s statistics and to adjust for it with the trim and fill method.

## 3. Results

### 3.1. Study Selection

**Figure 1:**
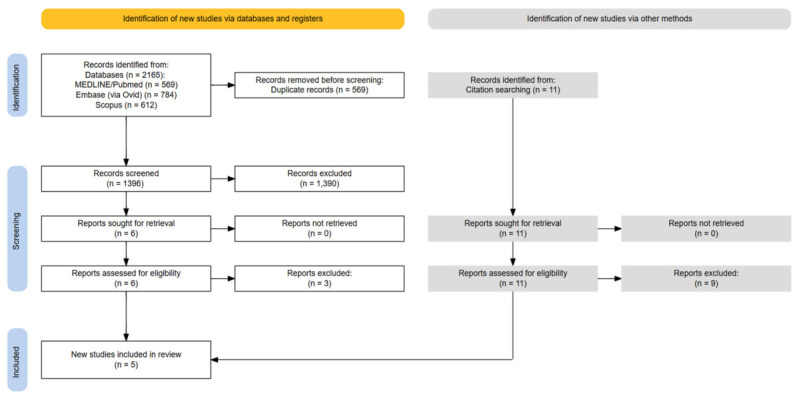
PRISMA flowchart for the study selection process.

Our search retrieved a total of 1414 records. Among them, 53 duplicates were identified and excluded; the remaining 1361 titles and abstracts were screened for relevance and six of them were deemed eligible for inclusion in the full-text review. Moreover, 11 records were identified through a manual search of the reference lists of the included articles or of relevant review articles on the same topic; all of them were retrieved in full text and were included in the following step of the selection process. A total of 17 studies underwent a full-text review and five of them [16,17,18,19,20] met the eligibility criteria and were included in the final review. A detailed flowchart of the study selection process according to PRISMA guidelines can be found in Figure 1. Table S5 in the Supplementary Materials lists the studies excluded during the full-text screening [21,22,23,24,25,26,27,28,29,30,31,32] and the reason for their exclusion.

### 3.2. Characteristics of Included Studies

We included five studies in our review [16,17,18,19,20]; detailed characteristics for each study are listed in Table 1. Two studies adopted a cohort design prospective [16,17] while two a retrospective cohort design [18,19], and one adopted a case–control design [20]. Overall, 3,703,877 subjects were included in the cohort studies; the case–control study included 400 participants. Publication years ranged from 2011 to 2024. Among studies providing enough information, participants’ mean age ranged between 56.3 and 76.4 years, and the proportion of female cases was 49.3%.

Among the cohort studies, Golimstok et al. [16] had a mean follow-up of 12.1 years and Dobrosavljevic et al. [18] had a median follow-up of 14.1 years; in Levine et al. [17] the maximum extent of follow-up was 17.2 years, and in Tzeng et al. [19] this was up to 10 years.

Data sources of the selected studies included hospital records, research databases, health maintenance organization records, population-based registries, national health databases, and multiple population-based registers.

### 3.3. Definition of Exposure and Outcome

To define the exposure (ADHD diagnosis), three studies [17,18,19] used ICD criteria, while two [16,20] used DSM criteria. Two studies [16,20] included patients with an ADHD diagnosis in pediatric age (even if retrospectively), two included diagnosis in adulthood [17,19], and one [18] did not pose restriction to age of diagnosis.

With reference to outcome definition, four studies [16,17,18,19] considered all-cause dementia, three AD [16,19,20], two DLB [16,20], two VaD [16,19], and one FTD [16].

Detailed characteristics for each study are described in Table 2.

### 3.4. Quality Appraisal

The potential risk of bias, quantified using the Newcastle–Ottawa scale, is shown in Table 3 both for cohort and case–control studies. The domains regarding selection and outcome were the most prone to bias. Overall, one study [20] had an overall score of 6, one of 7 [16], one of 8 [17] and two [18,19] of 9.

### 3.5. Association Between ADHD and Risk of Dementia

Studies exploring the association between ADHD and dementia from all causes provided HRs as measures of effect (see Table 4 for details).

**Figure 2:**
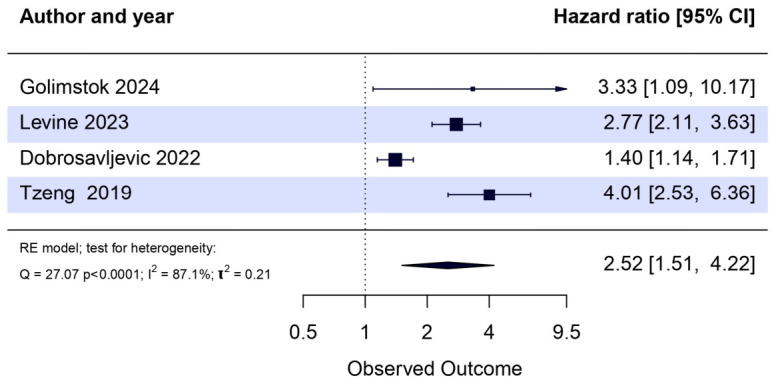
Forest plot for meta-analysis on the association between ADHD and all-cause dementia [16,17,18,19].

A meta-analysis was performed on four studies [16,17,18,19], for a total of 3,703,877 participants (11,071 with ADHD, 3,692,779 without ADHD); the pooled HR was 2.52 (95%CI 1.51–4.22, *p* < 0.001), showing a significant increase in the risk of dementia in subjects with ADHD (Figure 2). The I^2^ value we found was 87.7%, indicating very high heterogeneity.

Egger’s test (*p* = 0.358) and the funnel plot (Figure S1 in the Supplementary Materials) did not show significant risk of publication bias; however, their interpretation is severely impaired by the low number of included studies. Because of this issue, a conclusive evaluation of publication bias was not possible and for this reason the trim-and-fill method was not applied.

Figure S2 and Table S6 in the Supplementary Materials show the results of the leave-one-out analysis: the paper by Dobrosavljevic et al. [18] was the only to appear as an outlier and to disproportionately drive the overall pooled result. Thus, we performed a sensitivity analysis with exclusion of this study and we obtained a HR of 3.15 (95%CI 2.33–4.25, *p* < 0.001), with a drop in heterogeneity (I^2^ = 22.91%). See Figure S3 in the Supplementary Materials for the forest plot.

We also performed a meta-regression for the age of ADHD definition (diagnosis only in childhood vs. diagnosis in adulthood or at any age); we did not find a significant effect for this factor (*p* = 0.703).

For the association between ADHD and AD, we did not perform a meta-analysis, since only two studies provided ORs [16,20], whereas the other one provided a HR [19]. In none of the three studies the association had a significant effect size (see Table 4). Likewise, we did not perform a meta-analysis on studies regarding DLB and VaD, due to their low number. For DLB, both included studies provided measures of effect with significant values (OR 14.45, 95%CI 1.90–109.63 and OR 5.1, 95%CI 2.70–9.60), whereas this was true only for one of the two studies on VaD (HR 6.29 95%CI 2.71–25.85). The HRs and ORs of these studies and of the one regarding FTD are reported in Table 4.

## 4. Discussion

To our knowledge, this is the first systematic review to provide a meta-analysis on the association between ADHD and the risk of dementia. Our most relevant finding is the positive association between ADHD and all-cause dementia, which was observed in a population of more than 3 million people. This was the result of a coherent set of data, since all cohort studies provided significant HRs, although the strength of the association varied between them. This finding was confirmed also by the influence analysis and the removal of an influential study even increased the estimated HR (3.15 95%CI 2.33–4.25).

Different hypotheses can be formulated on the nature of the association we found, as reviewed by Callahan et al. [33], who provided a thorough discussion on the topic (although more oriented towards mild cognitive impairment rather than overt dementia). The first one is that ADHD and dementia represent two points along a single pathophysiological continuum, i.e., that neurodevelopmental alterations causing ADHD also predispose to cognitive disorders in later life; in this case, a true biological relationship would link these conditions.

The second one is that the association is mainly driven by the fact that people with ADHD have a higher prevalence of risk factors for dementia, like psychiatric disorders, cardiovascular comorbidities and lifestyle risk factors (smoking, substance use, obesity, physical inactivity) [34,35,36]; however, most of the HRs we included in our analysis were already adjusted for these factors and this reduces the concern.

The third hypothesis is that the relationship is overestimated because ADHD is a phenotypic mimic of dementia, i.e., because the two disorders share some clinical features, but are based on totally distinct mechanisms; in other terms, prodromal dementia, being characterized by executive dysfunction and attention deficit, could be misdiagnosed as adult-onset ADHD and this would create a spurious association between ADHD and dementia, which would be in reality the result of a reverse-causation effect. This is a relevant issue and most of the included studies tried to address it with different strategies: in two of them [16,17], a stratified analysis was performed with the exclusion of dementia diagnoses in the first years of the follow-up: in both cases, even if the longer was the initial lag time, the lower was the HR, and the association also remained significant for the longest lag time (12 or 15 years). One of these studies [16] also excluded subjects with early radiological markers of neurodegeneration to limit the risk of misdiagnosis. In another study [18], dementia cases with onset age earlier than 50 years were excluded, due to higher risk of misdiagnosis of ADHD, and post hoc analyses were performed to look for reverse causation, with negative result. It is also worth noting that the metaregression we conducted for the role of ADHD diagnosis in adulthood did not show a significant effect; moreover, the exclusion of cross-sectional studies should have limited the reverse causation issues. Thus, even if the phenotypic mimicry/reverse causation mechanism may have some impact on the relationship between ADHD and dementia, it does not seem the exclusive driver of this relationship.

None of the included studies showed a significant association between ADHD and AD. These results are quite surprising when considering the significant pooled HR we obtained for all-cause dementia, since AD is the most frequent type; a possible explanation can be proposed if one admits the aforementioned hypothesis of ADHD as a phenotypic mimic of dementia: if this were true, it could be thought that when considering a specific type of dementia, defined by stricter criteria, similarities with ADHD cognitive profile in later life were less evident, thus undermining the correlation observed for all-cause dementia. Our findings on AD are coherent with those of the previous reviews on the subject [4]; however, we should mention another review [7] which explored this relation taking into account also other types of studies, such as those based on genetic data, and provided a more complex picture. In particular, several studies showed an association between polygenic risk scores for ADHD and AD risk, but no association was observed in Mendelian randomization studies. How these results can be reconciled among them and with the observational studies we included in our review is unclear; the interaction between genetic and environmental risk factors could play a role in this context.

Even if a pooled estimate was not performed due to sample size issues, available data point out a significant association between ADHD and DLB. This is particularly interesting, since a relationship between ADHD and both DLB and Parkinson’s disease has been explored for a long time on epidemiological and theoretical grounds [37]. The main link among these three disorders has been identified in the dopaminergic system: even if the description of ADHD as a hypodopaminergic state is probably an oversimplification, converging genetic, metabolic and neurophysiological evidence suggest that a dopaminergic dysfunction can be found in this disorder, although its precise nature and role are far from being clarified, due to conflicting results [38]. Also noradrenaline has been deemed responsible for the potential association between DLB and ADHD, since previous studies have observed neuronal loss and altered functional connectivity in the noradrenergic locus coeruleus in the former disorder [39,40] and altered noradrenergic projections to the prefrontal cortex in the latter [41]. However, the role of neurotransmitters in the hypothetical connection between ADHD and DLB still remains speculative, since no direct empirical evidence is available on this topic.

Conflicting results were found for the influence of ADHD over the risk of developing VaD. However, our findings are still relevant, since they fill a gap regarding VaD that had been underlined in a previous review on the same topic [7]. Even if the limited number of retrieved studies prevented us from drawing definitive conclusions, there are some additional considerations in favor of an association between ADHD and VaD: the main one is that ADHD is associated with worse cardio- and cerebrovascular health and lower adherence to risk-reducing therapies [23,42], even if there are probably other mechanisms, since the study by Tzeng et al. [19] showed a significant effect size even after correcting for cardiovascular risk factors. We can also observe that VaD is the second most frequent cause of dementia after AD; for this reason, it can be supposed that the significant association we found for all-cause dementia can be driven by the one for VaD.

### Limitations

We acknowledge that this study is not without limitations. The main one is probably the heterogeneity in outcome definitions. Since most existing studies evaluated the impact of ADHD on overall dementia, grouping all types of dementia together was unavoidable, but it is clear that this can be problematic, since each type of dementia has a different pathobiology. Even for individual dementia types, definition issues can be easily found, with particular reference to the total lack of data on biological-based diagnostic criteria (AD is probably the main example from this point of view).

The definition of exposure was heterogeneous too, since some studies adopted the ICD codes for the diagnosis of ADHD, others the DSM diagnostic criteria; among studies using ICD, only one [19] provided information on validation procedures. Age at ADHD diagnosis is another issue related to exposure definition and its implications have been widely discussed above.

Heterogeneity also arose from different covariates used for adjustment in included articles; this poses the problem of possible residual confounding.

Moreover, it should be noted that in our meta-analysis, one study [17] accounted for more than 90% of all the included subjects; even if meta-analytical models are designed to handle studies with different sample sizes, such an extreme imbalance should be taken into account.

The limited number of included studies is another matter of concern, since it undermines the generalizability of our results and prevented us from performing more analyses, for example to assess a dose–response relationship or to explore the role of sex or ADHD medications as moderators of the ADHD–dementia association. The limited amount of available data was also the reason we did not perform a quantitative synthesis of evidence regarding each type of dementia separately. Moreover, the low number of included studies limited also the reliability of the publication bias analysis.

Furthermore, since intellectual disability could be a relevant comorbidity in ADHD, lack of information on cognitive testing at the time of ADHD diagnosis is a relevant issue. This problem is typical not only of ADHD, but of all neurodevelopmental disorders, including autism spectrum disorders (ASD). Also for this disease an increased risk of dementia has been observed [43], but distinguishing ASD features from those of dementia in adult individuals can be challenging and has prompted specific consensus reports [44].

## 5. Conclusions

In conclusion, our systematic review and meta-analysis shows that ADHD is associated with a significantly increased risk of all-cause dementia in later life; the impact of this disorder on single, specific types of dementia is less clear and deserves attention in future research. If forthcoming studies show that a real causal relationship drives the association between ADHD and dementia, early diagnosis and management of this disorder will play an increasing role in public health strategies for the elderly.

## Figures and Tables

**Table 1:** Characteristics of included studies.

Study	Country	Study Design	Participants, *n*	Age, Mean or Median (Years)	Female, *n* (%)	Data Source
Golimstok 2024 [16]	Argentina, Israel	Prospective cohort	161 exposed 109 not exposed	76.4	152 (56.3)	Hospital records
Levine 2023 [17]	Israel	Prospective cohort	730 exposed 108,488 not exposed	56.3	56,474 (51.7)	Health maintenance organization records
Dobrosavljevic 2022 [18]	Sweden	Retrospective cohort	9532 exposed 3,582,157 not exposed	63.0	1,770,605 (49.3)	Population-based registries
Tzeng 2019 [19]	Taiwan	Retrospective cohort	675 exposed 2025 not exposed	NA	744 (27.6)	National health databases
Golimstok 2011 [20]	Argentina	Case–control	251 cases 149 controls	74.2	338 (66.4)	Hospital records

NA: not applicable.

**Table 2:** Definition of exposure and outcome in included studies.

Study	Time of ADHD Diagnosis	ADHD Ascertainment Method	Dementia Type(s)
Golimstok 2024 [16]	Childhood (retrospectively) ^a^	DSM-IV-TR ^a^	Dementia from all causes AD, DLB, VaD, FTD
Levine 2023 [17]	Adulthood	ICD-9 (code 314) ICD-10 (code F90)	Dementia from all causes
Dobrosavljevic 2022 [18]	Any age	ICD-9 ICD-10 People with prescription of medications for ADHD	Dementia from all causes
Tzeng 2019 [19]	Adulthood	ICD-9-CM (code 314)	Dementia from all causes AD, VaD
Golimstok 2011 [20]	Childhood (retrospectively) ^a^	DSM-IV ^a^	AD, DLB

^a^ Presence of ADHD during childhood was retrospectively assessed through the use of Wender Utah Rating Scale (WURS). DSM-IV: Diagnostic and Statistical Manual of Mental Disorders, 4th edition; TR: text revision; ICD: International Classification of Diseases; CM: clinical modification; AD: Alzheimer’s disease; DLB: Lewy body dementia; VaD: vascular dementia; FTD: frontotemporal dementia.

**Table 3:** Risk of bias evaluation with Newcastle–Ottawa scale.

Study	Selection	Comparability	Outcome	Total Points
Golimstok 2024 [16]	3	1	3	7
Levine 2023 [17]	4	2	2	8
Dobrosavljevic 2022 [18]	4	2	3	9
Tzeng 2019 [19]	4	2	3	9
Golimstok 2011 [20]	2	2	2	6

**Table 4:** Associations between ADHD and dementia in included studies.

Dementia Type	Study	Effect Size	Covariates for Adjustment and/or Matching
Dementia from all causes	Golimstok 2024 [16]	OR 5.29 (1.80–15.59) HR 3.33 (1.09–10.17)	Age, depression (HR) None (OR)
	Levine 2023 [17]	HR 2.77 (2.11–3.63)	Age, sex, socioeconomic status, smoking status, diabetes, BMI/obesity, vascular comorbidities, psychostimulant medication use, depression, cardiovascular disorders, Parkinson’s disease, traumatic brain injury
	Dobrosavljevic 2022 [18]	HR 1.40 (1.15–1.72)	Age, sex, education, diabetes, BMI/obesity, vascular comorbidities, metabolic disorders, psychiatric disorders, other developmental disorders
	Tzeng 2019 [19]	HR 4.01 (2.53–6.36)	Age, sex, education, place of residence, diabetes, cardiovascular disorders, stroke, traumatic brain injury, metabolic disorders, psychiatric disorders, other developmental disorders
AD	Golimstok 2024 [16]	OR 2.75 (0.30–24.96)	None
	Tzeng 2019 [19]	HR 0.52 (0.06–4.53)	Age, sex, education, place of residence, diabetes, cardiovascular disorders, stroke, traumatic brain injury, metabolic disorders, psychiatric disorders, other developmental disorders
	Golimstok 2011 [20]	OR 1.10 (0.70–1.50)	None
DLB	Golimstok 2024 [16]	HR 54.55 (7.49–397.51) OR 14.45 (1.90–109.63)	Sex, age, education, depression (HR) None (OR)
	Golimstok 2011 [20]	OR 5.1 (2.70–9.60)	None
VaD	Golimstok 2024 [16]	OR 1.02 (0.17–6.18)	None
	Tzeng 2019 [19]	HR 6.29 (2.71–25.85)	Age, sex, education, place of residence, diabetes, cardiovascular disorders, stroke, traumatic brain injury, metabolic disorders, psychiatric disorders, other developmental disorders
FTD	Golimstok 2024 [16]	OR 2.05 (0.08–50.71)	None

OR: odds ratio; HR: hazard ratio; AD: Alzheimer’s disease; DLB: Lewy body dementia; VaD: vascular dementia; FTD: frontotemporal dementia.

## Data Availability

No new data were created or analyzed in this study. Data sharing is not applicable to this article.

## Supplementary Materials

The following supporting information can be downloaded at https://www.mdpi.com/article/10.3390/brainsci16060646/s1: Table S1. PRISMA flowchart; Table S2. PubMed/MEDLINE search history; Table S3. Scopus search history; Table S4. Embase (via Ovid) search history; Table S5. Characteristics of excluded studies; Table S6. Influence analysis for all-cause dementia. Figure S1. All-cause dementia funnel plot (boundary lines represent the 95% confidence intervals around the pooled effect estimate); Figure S2. Influence analysis or all-cause dementia; Figure S3. Sensitivity analysis for all-cause dementia.
