# Supplementary material for: Attention Deficit/Hyperactivity Disorder and Risk of Dementia: A Systematic Review and Meta-Analysis
Source: Brain Sci. 2026 Jun 18;16(6):646. doi: 10.3390/brainsci16060646 (PMC13297260; doi:10.3390/brainsci16060646)
Supplement: Supplementary file 1 [file brainsci-16-00646-s001.zip › Table S2.pdf]

Table S2. PubMed/MEDLINE Search History

**Search**	**PubMed/MEDLINE Query – 17th May, 2025**	**Items found**
#18	#1 AND #17	569
#17	#4 OR #5 OR #7 OR #15 OR #16	254396
#16	#12 AND #14	3229
#15	#12 AND #13	27344
#14	#9 OR #10	7088
#13	#6 OR #8 OR #11	156032
#12	#2 OR #3	396380
#11	"Parkinson Disease"[Mesh] OR “Parkinson Disease*"[tiab] OR “Parkinson’s Disease*"[tiab] OR “Paralysis Agitans"[tiab] OR “Primary Parkinsonism"[tiab]	149057
#10	"Corticobasal Degeneration"[Mesh] OR "Cortico-basal Degeneration*"[tiab] OR "Cortico basal Degeneration*"[tiab] OR "Corticobasal Degeneration*"[tiab] OR "Cortico-basal Syndrome*"[tiab] OR "Cortico basal Syndrome*"[tiab] OR "Corticobasal Syndrome*"[tiab] OR "Cortico-basal Ganglionic Degeneration*"[tiab] OR "Cortico basal Ganglionic Degeneration*"[tiab] OR "Corticobasal Ganglionic Degeneration*"[tiab]	2752

#9	"Supranuclear Palsy, Progressive"[Mesh] OR “Progressive Supranuclear Pals*"[tiab] OR “Supranuclear Progressive Pals*"[tiab] OR “Richardson's Syndrome"[tiab] OR “Richardson Syndrome"[tiab] OR “Steele-Richardson-Olszewski Disease"[tiab] OR “Steele Richardson Olszewski Disease"[tiab] OR “Steele-Richardson-Olszewski Syndrome"[tiab] OR “Steele Richardson Olszewski Syndrome"[tiab] OR “Progressive Supranuclear Ophtalmoplegia"[tiab] OR “Supranuclear Progressive Ophtalmoplegia"[tiab]	5986
#8	"Multiple System Atrophy"[Mesh] OR “Multiple System Atroph*"[tiab] OR “Multisystem Atroph*"[tiab] OR “Multisystemic Atroph*"[tiab] OR “Multiple System Atrophy Syndrome"[tiab]	7219
#7	"Dementia, Vascular"[Mesh] OR "Vascular Dementia*"[tiab] OR "Arteriosclerotic Dementia*"[tiab] OR "Arteriosclerotic Encephalopath*"[tiab] OR "Binswanger Disease*"[tiab] OR "Binswanger Encephalopath*"[tiab] OR "Binswanger’s Disease*"[tiab] OR "Binswanger’s Encephalopath*"[tiab] OR "Chronic Progressive Subcortical Encephalopath*"[tiab] OR "Subcortical Leukoencephalopath*"[tiab]	13133
#6	"Lewy Body Disease"[Mesh] OR “Lewy Body Disease*"[tiab] OR “Lewy Body Type Senile Dementia*"[tiab] OR “Lewy Body Dementia*"[tiab]	6862
#5	“Frontotemporal Dementia"[Mesh] OR "Frontotemporal lobar degenerat*" [tiab] OR "Frontotemporal degenerat*"[tiab] OR "Frontotemporal dementia*"[tiab] OR "Frontotemporal lobe dementia*"[tiab] OR “Semantic dementia*” [tiab] OR “Multiple System Tauopathy with Presenile Dementia"[tiab] OR “Disinhibition-Dementia-Parkinsonism*"[tiab] OR “Hereditary Dysphasic Disinhibition Dementia"[tiab] OR “Pick's Disease*"[tiab] OR “Pick Disease*"[tiab] OR “Wilhelmsen-Lynch Disease*"[tiab] OR “FTLD*”[tiab] OR “FTD”[tiab] OR “FTDs”[tiab] OR “DDPAC"[tiab] OR “HDDD1"[tiab] OR “HDDD2"[tiab]	18710

#4	"Alzheimer Disease"[Mesh] OR “Alzheimer’s Disease*”[tiab] OR “Alzheimer Disease*”[tiab] OR “Alzheimer’s Syndrome*”[tiab] OR “Alzheimer Syndrome*”[tiab] OR “Alzheimer-Type Dementia*”[tiab] OR “Alzheimer Type Dementia*”[tiab] OR “Alzheimer Dementia*”[tiab] OR “Alzheimer’s Dementia*”[tiab] OR “Senile Dementia”[tiab] OR “Primary Senile Degenerative Dementia”[tiab] OR “Alzheimer’s Sclerosis”[tiab] OR “Alzheimer Sclerosis”[tiab] OR “Presenile Dementia*”[tiab]	221592
#3	"Cognitive Dysfunction"[Mesh] OR “Cognitive Dysfunction*”[tiab] OR “Cognitive Disorder*”[tiab] OR “Cognitive Impairment*”[tiab] OR “Cognitive Decline*”[tiab] OR “Mental Deterioration*”[tiab]	167565
#2	"Dementia"[Mesh] OR “Dementia*”[tiab] OR “Amentia*”[tiab]	291781
#1	"Attention Deficit Disorder with Hyperactivity"[Mesh] OR “ADHD”[tiab] OR “ADDH”[tiab] OR “Attention Deficit Disorders with Hyperactivity”[tiab] OR “Attention Deficit Hyperactivity Disorder*”[tiab] OR “Attention Deficit-Hyperactivity Disorder*”[tiab] OR “Attention Deficit/Hyperactivity Disorder*”[tiab] OR “Hyperkinetic Syndrome”[tiab] OR “Attention Deficit Disorder*”[tiab] OR “Minimal Brain Dysfunction”[tiab]	54385
