# Supplementary material for: Attention Deficit/Hyperactivity Disorder and Risk of Dementia: A Systematic Review and Meta-Analysis
Source: Brain Sci. 2026 Jun 18;16(6):646. doi: 10.3390/brainsci16060646 (PMC13297260; doi:10.3390/brainsci16060646)
Supplement: Supplementary file 1 [file brainsci-16-00646-s001.zip › Table S3.pdf]

Table S3. Scopus Search History

Search	Scopus Query – 17th May, 2025	Items found
18	1 AND 17	612
17	4 OR 5 OR 7 OR 15 OR 16	263328
16	12 AND 14	2569
15	12 AND 13	20173
14	9 OR 10	7183
13	6 OR 8 OR 11	162551
12	2 OR 3	328618
11	TITLE-ABS(“Parkinson Disease*”) OR TITLE-ABS(“Parkinson’s Disease*”) OR TITLE-ABS(“Paralysis Agitans”) OR TITLE-ABS(“Primary Parkinsonism”)	157611
10	TITLE-ABS("Cortico-basal Degeneration*”) OR TITLE-ABS(“Cortico basal Degeneration*”) OR TITLE-ABS(“Corticobasal Degeneration*”) OR TITLE-ABS(“Cortico-basal Syndrome*”) OR TITLE-ABS(“Cortico basal Syndrome*”) OR TITLE-ABS(“Corticobasal Syndrome*”) OR TITLE-ABS(“Cortico-basal Ganglionic Degeneration*”) OR TITLE-ABS(“Cortico basal Ganglionic Degeneration*”) OR TITLE-ABS(“Corticobasal Ganglionic Degeneration*”)	2950

9	TITLE-ABS(“Progressive Supranuclear Pals*”) OR TITLE-ABS(“Supranuclear Progressive Pals*”) OR TITLE-ABS(“Richardson's Syndrome”) OR TITLE-ABS(“Richardson Syndrome”) OR TITLE-ABS(“Steele-Richardson-Olszewski Disease”) OR TITLE-ABS(“Steele Richardson Olszewski Disease”) OR TITLE-ABS(“Steele-Richardson-Olszewski Syndrome”) OR TITLE-ABS(“Steele Richardson Olszewski Syndrome”) OR TITLE-ABS(“Progressive Supranuclear Ophtalmoplegia”) OR TITLE-ABS(“Supranuclear Progressive Ophtalmoplegia”)	5972
8	TITLE-ABS(“Multiple System Atroph*”) OR TITLE-ABS(“Multisystem Atroph*”) OR TITLE-ABS(“Multisystemic Atroph*”) OR TITLE-ABS(“Multiple System Atrophy Syndrome”)	6541
7	TITLE-ABS("Vascular Dementia*”) OR TITLE-ABS(“Arteriosclerotic Dementia*”) OR TITLE-ABS(“Arteriosclerotic Encephalopath*”) OR TITLE-ABS(“Binswanger Disease*”) OR TITLE-ABS(“Binswanger Encephalopath*”) OR TITLE-ABS(“Binswanger’s Disease*”) OR TITLE-ABS(“Binswanger’s Encephalopath*”) OR TITLE-ABS(“Chronic Progressive Subcortical Encephalopath*”) OR TITLE-ABS(“Subcortical Leukoencephalopath*”)	10877
6	TITLE-ABS(“Lewy Body Disease*”) OR TITLE-ABS(“Lewy Body Type Senile Dementia*”) OR TITLE-ABS(“Lewy Body Dementia*”)	3821
5	TITLE-ABS("Frontotemporal lobar degenerat*") OR TITLE-ABS(“Frontotemporal degenerat*”) OR TITLE-ABS(“Frontotemporal dementia*”) OR TITLE-ABS(“Frontotemporal lobe dementia*”) OR TITLE-ABS(“Semantic dementia*”) OR TITLE-ABS(“Multiple System Tauopathy with Presenile Dementia”) OR TITLE-ABS(“Disinhibition-Dementia-Parkinsonism*”) OR TITLE-ABS(“Hereditary Dysphasic Disinhibition Dementia”) OR TITLE-ABS(“Pick's Disease*”) OR TITLE-ABS(“Pick Disease*”) OR TITLE-ABS(“Wilhelmsen-Lynch Disease*”) OR TITLE-ABS(“FTLD*”) OR TITLE-ABS(“FTD”) OR TITLE-ABS(“FTDs”) OR TITLE-ABS(“DDPAC”) OR TITLE-ABS(“HDDD1”) OR TITLE-ABS(“HDDD2”)	19746

4	TITLE-ABS(“Alzheimer’s Disease*”) OR TITLE-ABS(“Alzheimer Disease*”) OR TITLE-ABS(“Alzheimer’s Syndrome*”) OR TITLE-ABS(“Alzheimer Syndrome*”) OR TITLE-ABS(“Alzheimer-Type Dementia*”) OR TITLE-ABS(“Alzheimer Type Dementia*”) OR TITLE-ABS(“Alzheimer Dementia*”) OR TITLE-ABS(“Alzheimer’s Dementia*”) OR TITLE-ABS(“Senile Dementia”) OR TITLE-ABS(“Primary Senile Degenerative Dementia”) OR TITLE-ABS(“Alzheimer’s Sclerosis”) OR TITLE-ABS(“Alzheimer Sclerosis”) OR TITLE-ABS(“Presenile Dementia*”)	232025
3	TITLE-ABS(“Cognitive Dysfunction*”) OR TITLE-ABS(“Cognitive Disorder*”) OR TITLE-ABS(“Cognitive Impairment*”) OR TITLE-ABS(“Cognitive Decline*”) OR TITLE-ABS(“Mental Deterioration*”)	181415
2	TITLE-ABS(“Dementia*”) OR TITLE-ABS(“Amentia*”)	193760
1	TITLE-ABS(“ADHD”) OR TITLE-ABS(“ADDH”) OR TITLE-ABS(“Attention Deficit Disorders with Hyperactivity”) OR TITLE-ABS(“Attention Deficit Hyperactivity Disorder*”) OR TITLE-ABS(“Attention Deficit-Hyperactivity Disorder*”) OR TITLE-ABS(“Attention Deficit/Hyperactivity Disorder*”) OR TITLE-ABS(“Hyperkinetic Syndrome”) OR TITLE-ABS(“Attention Deficit Disorder*”) OR TITLE-ABS(“Minimal Brain Dysfunction”)	60952
