# Supplementary material for: Attention Deficit/Hyperactivity Disorder and Risk of Dementia: A Systematic Review and Meta-Analysis
Source: Brain Sci. 2026 Jun 18;16(6):646. doi: 10.3390/brainsci16060646 (PMC13297260; doi:10.3390/brainsci16060646)
Supplement: Supplementary file 1 [file brainsci-16-00646-s001.zip › Table S4.pdf]

Table S4. Embase (via OVID) Search History

Search	Embase (via OVID) Query – 17th May, 2025	Items found
21	19 NOT 20	784
20	Limit 19 to conference abstract status	262
19	Limit 18 to "remove medline records"	1046
18	1 AND 17	2663
17	4 OR 5 OR 7 OR 15 OR 16	394960
16	12 AND 14	8167
15	12 AND 13	77398
14	9 OR 10	13074
13	6 OR 8 OR 11	257613
12	2 OR 3	759727
11	exp Parkinson disease/ OR “Parkinson Disease*”.ti,ab,kf. OR “Parkinson’s Disease*”.ti,ab,kf. OR “Paralysis Agitans”.ti,ab,kf. OR “Primary Parkinsonism”.ti,ab,kf.	246259

10	exp Corticobasal degeneration/ OR "Cortico-basal Degeneration*".ti,ab,kf. OR "Cortico basal Degeneration*".ti,ab,kf. OR "Corticobasal Degeneration*".ti,ab,kf. OR "Cortico-basal Syndrome*".ti,ab,kf. OR "Cortico basal Syndrome*".ti,ab,kf. OR "Corticobasal Syndrome*".ti,ab,kf. OR "Cortico-basal Ganglionic Degeneration*".ti,ab,kf. OR "Cortico basal Ganglionic Degeneration*".ti,ab,kf. OR "Corticobasal Ganglionic Degeneration*".ti,ab,kf.	5584
9	exp Progressive supranuclear palsy/ OR "Progressive Supranuclear Pals*".ti,ab,kf. OR "Supranuclear Progressive Pals*".ti,ab,kf. OR "Richardson's Syndrome".ti,ab,kf. OR "Richardson Syndrome".ti,ab,kf. OR "Steele-Richardson-Olszewski Disease".ti,ab,kf. OR "Steele Richardson Olszewski Disease".ti,ab,kf. OR "Steele-Richardson-Olszewski Syndrome".ti,ab,kf. OR "Steele Richardson Olszewski Syndrome".ti,ab,kf. OR "Progressive Supranuclear Ophtalmoplegia".ti,ab,kf. OR "Supranuclear Progressive Ophtalmoplegia".ti,ab,kf.	11006
8	exp Multiple system atrophy/ OR "Multiple System Atroph*".ti,ab,kf. OR "Multisystem Atroph*".ti,ab,kf. OR "Multisystemic Atroph*".ti,ab,kf. OR "Multiple System Atrophy Syndrome".ti,ab,kf.	11656
7	exp Multiinfarct dementia/ OR "Vascular Dementia*".ti,ab,kf. OR "Arteriosclerotic Dementia*".ti,ab,kf. OR "Arteriosclerotic Encephalopath*".ti,ab,kf. OR "Binswanger Disease*".ti,ab,kf. OR "Binswanger Encephalopath*".ti,ab,kf. OR "Binswanger's Disease*".ti,ab,kf. OR "Binswanger's Encephalopath*".ti,ab,kf. OR "Chronic Progressive Subcortical Encephalopath*".ti,ab,kf. OR "Subcortical Leukoencephalopath*".ti,ab,kf.	19676
6	exp Diffuse Lewy body disease/ OR "Lewy Body Disease*".ti,ab,kf. OR "Lewy Body Type Senile Dementia*".ti,ab,kf. OR "Lewy Body Dementia*".ti,ab,kf.	14958

5	exp Frontotemporal dementia/ OR "Frontotemporal lobar degenerat*".ti,ab,kf. OR "Frontotemporal degenerat*".ti,ab,kf. OR "Frontotemporal dementia*".ti,ab,kf. OR "Frontotemporal lobe dementia*".ti,ab,kf. OR “Semantic dementia*”.ti,ab,kf. OR “Multiple System Tauopathy with Presenile Dementia".ti,ab,kf. OR “Disinhibition-Dementia-Parkinsonism*".ti,ab,kf. OR “Hereditary Dysphasic Disinhibition Dementia".ti,ab,kf. OR “Pick's Disease*".ti,ab,kf. OR “Pick Disease*".ti,ab,kf. OR “Wilhelmsen-Lynch Disease*".ti,ab,kf. OR “FTLD*”.ti,ab,kf. OR “FTD”.ti,ab,kf. OR “FTDs”.ti,ab,kf. OR “DDPAC".ti,ab,kf. OR “HDDD1".ti,ab,kf. OR “HDDD2".ti,ab,kf.	34681
4	exp Alzheimer disease/ OR “Alzheimer’s Disease*”.ti,ab,kf. OR “Alzheimer Disease*”.ti,ab,kf. OR “Alzheimer’s Syndrome*”.ti,ab,kf. OR “Alzheimer Syndrome*”.ti,ab,kf. OR “Alzheimer-Type Dementia*”.ti,ab,kf. OR “Alzheimer Type Dementia*”.ti,ab,kf. OR “Alzheimer Dementia*”.ti,ab,kf. OR “Alzheimer’s Dementia*”.ti,ab,kf. OR “Senile Dementia”.ti,ab,kf. OR “Primary Senile Degenerative Dementia”.ti,ab,kf. OR “Alzheimer’s Sclerosis”.ti,ab,kf. OR “Alzheimer Sclerosis”.ti,ab,kf. OR “Presenile Dementia*”.ti,ab,kf.	334167
3	exp Cognitive defect/OR “Cognitive Dysfunction*”.ti,ab,kf. OR “Cognitive Disorder*”.ti,ab,kf. OR “Cognitive Impairment*”.ti,ab,kf. OR “Cognitive Decline*”.ti,ab,kf. OR “Mental Deterioration*”.ti,ab,kf.	737261
2	exp Dementia/ OR “Dementia*”.ti,ab,kf. OR “Amentia*”.ti,ab,kf.	536716
1	exp Attention deficit hyperactivity disorder/ OR “ADHD”.ti,ab,kf. OR “ADDH”.ti,ab,kf. OR “Attention Deficit Disorders with Hyperactivity”.ti,ab,kf. OR “Attention Deficit Hyperactivity Disorder*”.ti,ab,kf. OR “Attention Deficit-Hyperactivity Disorder*”.ti,ab,kf. OR “Attention Deficit/Hyperactivity Disorder*”.ti,ab,kf. OR “Hyperkinetic Syndrome”.ti,ab,kf. OR “Attention Deficit Disorder*”.ti,ab,kf. OR “Minimal Brain Dysfunction”.ti,ab,kf.	96886
