# Supplementary material for: Attention Deficit/Hyperactivity Disorder and Risk of Dementia: A Systematic Review and Meta-Analysis
Source: Brain Sci. 2026 Jun 18;16(6):646. doi: 10.3390/brainsci16060646 (PMC13297260; doi:10.3390/brainsci16060646)
Supplement: Supplementary file 1 [file brainsci-16-00646-s001.zip › Table S5.pdf]

Table S5. Characteristics of excluded studies

Article	Reason for exclusion
Libutzki 2024 [21]	Inadequate study design (cross-sectional)
Callahan 2022 [22]	Inadequate outcome definition (neuropsychological indices rather than dementia)
Garcia-Argibay 2022 [23]	Inadequate exposure definition (genetic liability to ADHD rather than ADHD itself)
Pagoni 2022 [24]	Inadequate exposure definition (genetic liability to ADHD rather than ADHD itself)
Wei 2022 [25]	Inadequate study design (Mendelian randomization rather than longitudinal)
Zhang 2022 [26]	Inadequate study design (multi-generation cohort study)
Callahan 2021 [27]	No results about correlation between ADHD and dementia (study on the role of vascular risk factors ad moderators of this correlation)
Du Rietz 2021 [28]	Inadequate study design (cross-sectional)
Fan 2020 [29]	Inadequate exposure definition (Parkinson’s disease rather than dementia)
Curtin 2018 [30]	No results specific for dementia (diseases of the basal ganglia and cerebellum rather than dementia)
Fluegge 2018 [31]	Inadequate exposure definition (percentage of psychiatric hospitalizations due to ADHD rather than ADHD itself)

Ivanchak 2011 [32]	Inadequate study design (cross-sectional)
