# Supplementary material for: Attention Deficit/Hyperactivity Disorder and Risk of Dementia: A Systematic Review and Meta-Analysis
Source: Brain Sci. 2026 Jun 18;16(6):646. doi: 10.3390/brainsci16060646 (PMC13297260; doi:10.3390/brainsci16060646)
Supplement: Supplementary file 1 [file brainsci-16-00646-s001.zip › Table S6.pdf]

Table S6. Influence analysis

Study	Externally standardized residuals	DFFITS values	Cook's distance	Covariance ratios	τ^2	Residual heterogeniety	Hat values	Weights	DFBETAS values	Is influent[a]
Golimstok 2024 [16]	0.3819	0.1324	0.0191	1.3783	0.2576	26.1947	0.1289	12.8854	0.1269	No
Levine 2023 [17]	0.1536	0.0448	0.0031	2.0664	0.3318	18.2165	0.3002	30.0166	0.0457	No
Dobrosavljevic 2022 [18]	-3.5311	-2.3226	0.7177	0.3401	0.0188	1.8519	0.3119	31.1870	-1.9474	Yes
Tzeng 2019 [19]	1.1404	0.7004	0.4233	1.1511	0.1738	16.8562	0.2591	25.9110	0.7035	No

[a] A study was considered influent when at least one of the following requirements is met:

- the absolute DFFITS value is larger than 3 p/(k-p)3*(p/(k-p)), where p is the number of model coefficients and k the number of cases
- the lower tail area of a chi-square distribution with p degrees of freedom cut off by the Cook's distance is larger than 50%
- the hat value is larger than 3 (p/k)3*(p/k)
- any DFBETAS value is larger than 1
