# Supplementary figures and images for: Attention Deficit/Hyperactivity Disorder and Risk of Dementia: A Systematic Review and Meta-Analysis
Source: Brain Sci. 2026 Jun 18;16(6):646. doi: 10.3390/brainsci16060646 (PMC13297260; doi:10.3390/brainsci16060646)

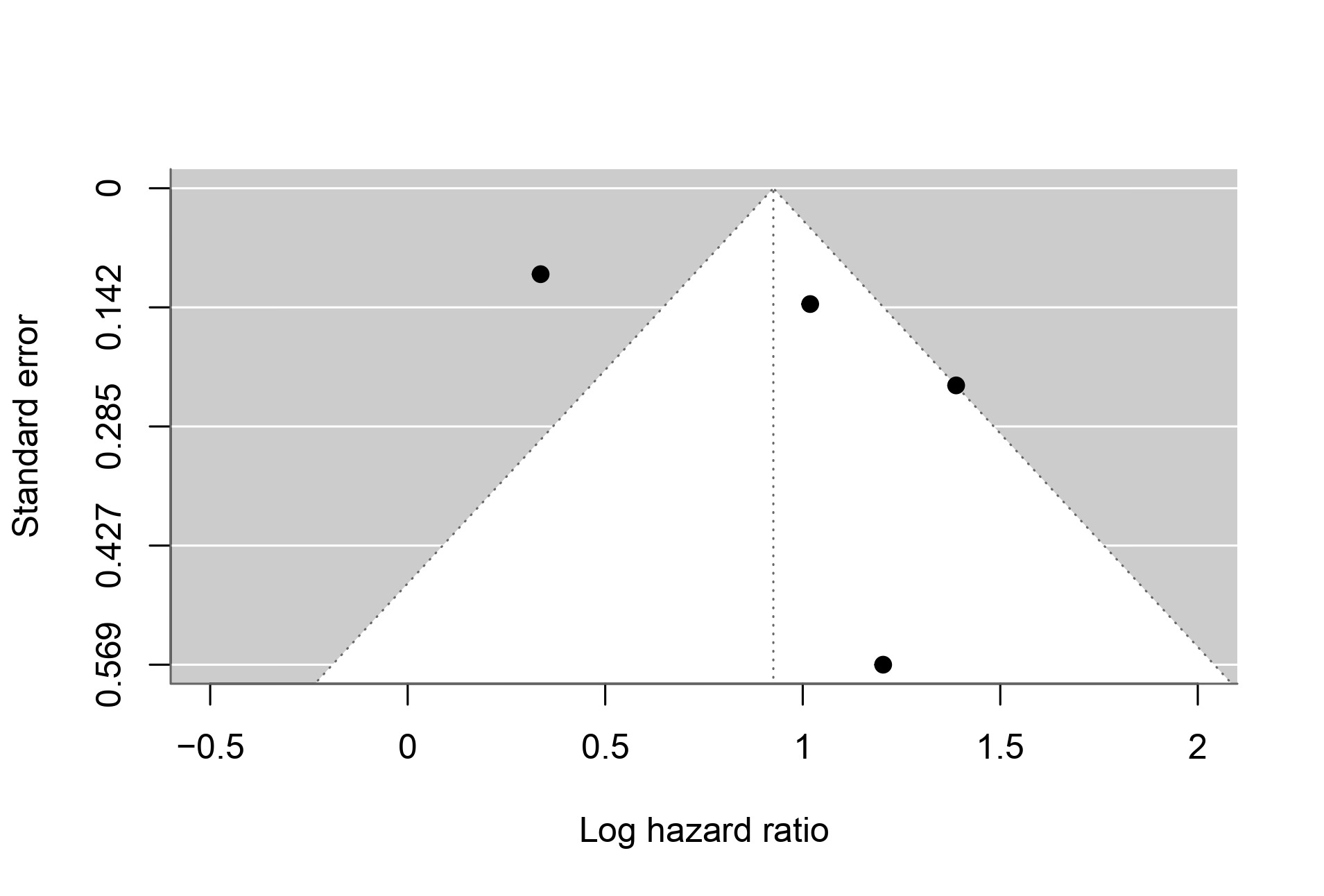

Supplement: Supplementary file 1 [file brainsci-16-00646-s001.zip › Figure S1.jpg]

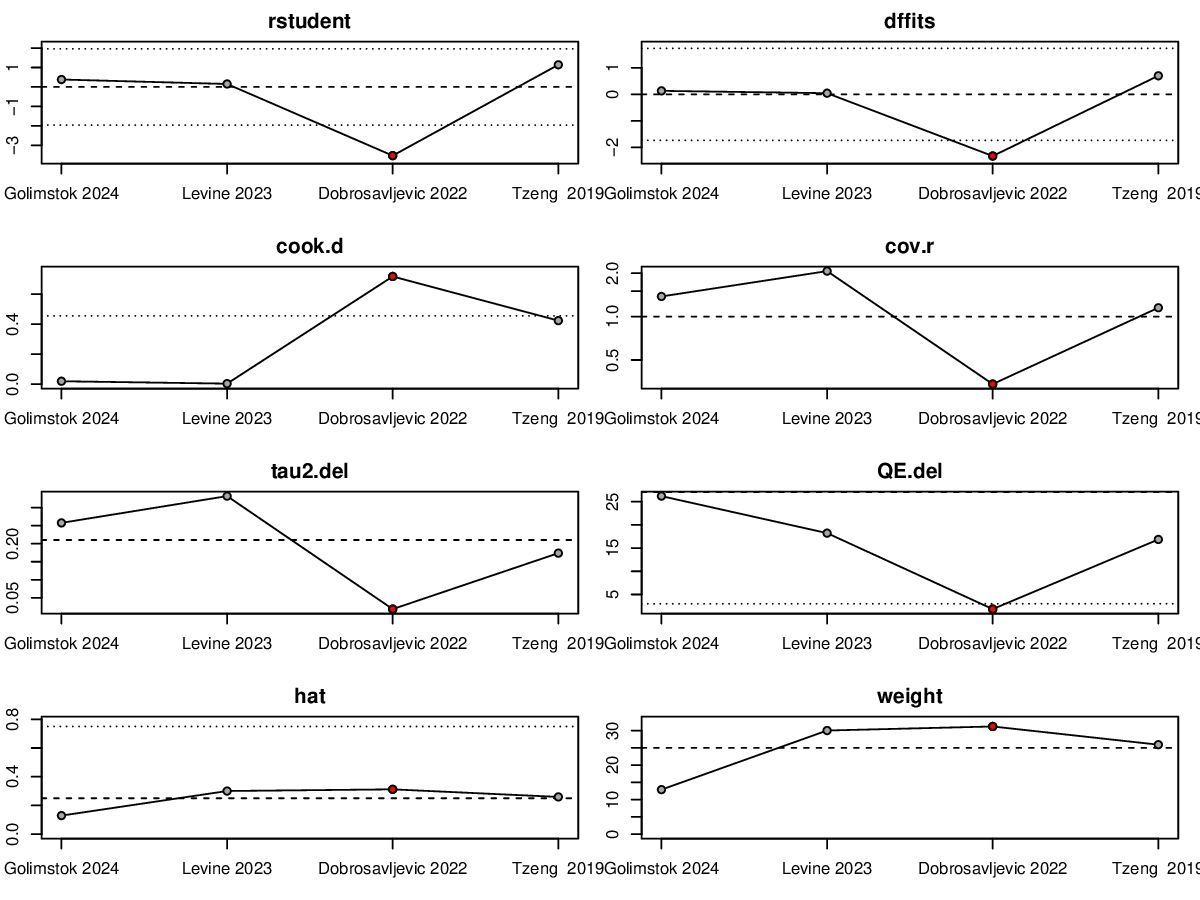

Supplement: Supplementary file 1 [file brainsci-16-00646-s001.zip › Figure S2.jpg]

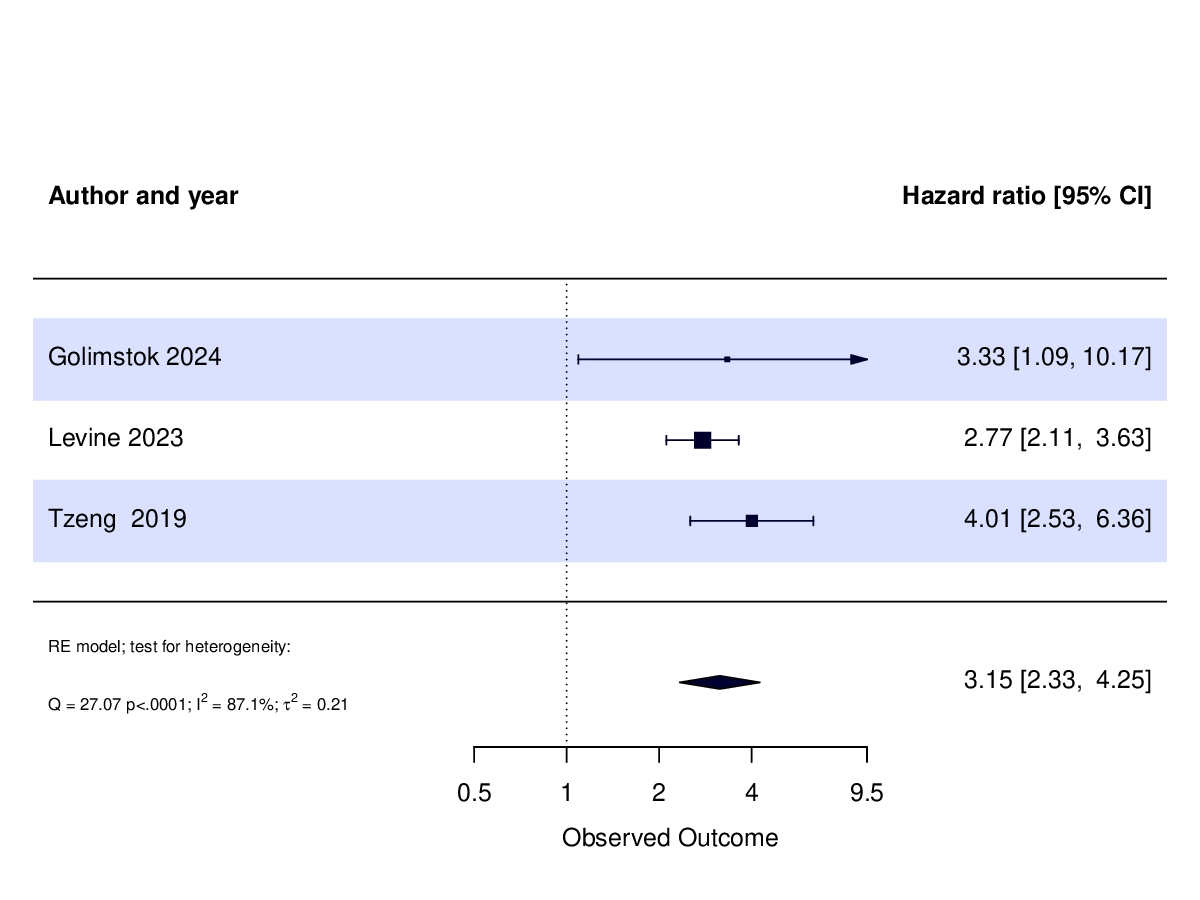

Supplement: Supplementary file 1 [file brainsci-16-00646-s001.zip › Figure S3.jpg]
